# Autophagy Deficits Underly Selective Neuronal Vulnerability to Alpha Synuclein Fibrils in Alzheimer’s and Parkinson’s iPSC Derived Neurons

**DOI:** 10.1002/alz.087271

**Published:** 2025-01-03

**Authors:** Ajantha S Abey, Bryan Ng, Rachel Heon‐Roberts, Becky C. Carlyle, Nora Bengoa‐Vergniory, Richard Wade‐Martins

**Affiliations:** ^1^ University of Oxford, Oxford United Kingdom; ^2^ Agency for Science, Technology and Research, Singapore Singapore; ^3^ Achucarro Basque Centre for Neuroscience, Leioa Spain; ^4^ Oxford Parkinson’s Disease Centre, Oxford United Kingdom

## Abstract

**Background:**

Alzheimer’s (AD) and Parkinson’s disease (PD) feature progressive neurodegeneration in a remarkably regionally selective manner. *Post mortem* studies have posited a role for cell autonomous mechanisms driving this, so we aimed to examine a live human induced pluripotent stem cell (iPSC) model to see whether it can replicate the phenomenon of selective neuronal vulnerability, so to better determine disease mechanisms and therapeutic targets.

**Method:**

iPSC‐derived neurons offer a rare opportunity to examine cell autonomous vulnerability in live human cells. iPSCs from patients with AD‐related presenilin‐1 mutations (n = 6), PD‐related leucine rich repeat kinase 2 mutations (n = 6), and isogenic corrected (n = 4) and healthy controls (n = 4) have been differentiated into both cortical and midbrain dopaminergic neurons to enable comparison of pre‐formed fibril induced pathology in different neuronal subtypes from the same patient. We then examined lysosomal number, morphology, degradation, pH, and calcium using live imaging assays, alongside mitochondrial biology, and electrophysiology to understand underlying drivers of vulnerability in the cell types.

**Result:**

Upon insult with alpha‐synuclein PFFs, AD and PD dopaminergic neurons produce substantial Lewy‐like pathology, whereas cortical neurons remain relatively resilient to alpha‐synuclein aggregation, suggesting cell‐type vulnerability. PSEN1‐Intron‐4‐Deletion cortical neurons, however, had significantly elevated pathology. These lines displayed hyperactivity on microelectrode arrays and abnormal lysosomal biology, including increased LAMP1 and dysregulated calcium. PFF‐insulted AD cortical neurons also have impaired neurite outgrowth, while PD cortical neurons are resilient.

**Conclusion:**

These preliminary results show relative vulnerability of AD against PD cortical neurons, and dopaminergic against cortical neurons to alpha‐synuclein aggregates for the first time. These suggest the selective vulnerability to proteinopathy in these diseases is reflected by the iPSC neuronal model and support the notion that cell intrinsic factors like autophagy drive vulnerability.

